# Pituitary Adenoma Surgery Survey: Neurosurgical Centers and Pituitary Adenomas

**DOI:** 10.1155/2022/7206713

**Published:** 2022-04-11

**Authors:** David Netuka, Andre Grotenhuis, Nicolas Foroglou, Francesco Zenga, Sebastien Froehlich, Florian Ringel, Nicolas Sampron, Nick Thomas, Martin Komarc, Martin Majovsky

**Affiliations:** ^1^Department of Neurosurgery and Neurooncology, First Faculty of Medicine, Charles University and Military University Hospital, Prague, Czech Republic; ^2^Department of Neurosurgery, Radboud University Medical Centre Nijmegen, Nijmegen, Netherlands; ^3^1st Department of Neurosurgery, Aristotle University of Thessaloniki, Thessaloniki, Greece; ^4^Department of Neuroscience “Rita Levi Montalcini”, Neurosurgery Unit, University of Turin, Turin, Italy; ^5^Department of Neurosurgery, Lariboisiere University Hospital, Paris, France; ^6^Department of Neurosurgery, Johannes Gutenberg-Universitat Mainz, Mainz, Germany; ^7^Neurosurgery Department, University Hospital Donostia, San Sebastian, Donostia, Spain; ^8^Department of Neurosurgery, Kings College, London, UK; ^9^Institute of Biophysics and Informatics, First Faculty of Medicine, Charles University, Prague, Czech Republic; ^10^Department of Methodology, Faculty of Physical Education and Sport, Charles University, Prague, Czech Republic

## Abstract

**Objective:**

Pituitary adenoma surgery has evolved rapidly in recent decades. This study aims to determine current practice across a wide range of European neurosurgical centers.

**Methods:**

A list of eligible departments performing pituitary adenoma surgery was created. The survey consisted of 58 questions. For analysis, the departments were divided into four subgroups: academic/nonacademic, high-volume/low-volume, “mainly endoscopic/mainly microscopic practice,” and geographical regions.

**Results:**

Data from 254 departments from 34 countries were obtained. In 108 centers (42.5%), <30 pituitary adenomas were operated per year. Twenty (7.9%) centers performed >100 adenoma surgeries per year. Number of neurosurgeons performing endonasal surgeries are as follows: 1 in 24.9% of centers and 2 in 49.8% of centers. All residents assisted endonasal surgeries in 126 centers (49.8%). In 28 centers (21.1%), all residents performed endonasal surgery under supervision during residency. In 141 centers (56.8%), the endoscopic approach was used in >90% of the surgeries. Regular pituitary board (either weekly or once a month) meetings were held in 147 centers (56.3%). Nonfunctioning adenomas represent >70% of pituitary caseload in 149 centers (58.7%).

**Conclusions:**

In our survey, most centers perform less than 100 surgeries for pituitary adenomas. In most centers, pituitary surgeries are performed by one or two neurosurgeons. Residents have a limited exposure to this type of surgery, and the formal pituitary board is not a standard. Nonfunctioning adenomas make up most of surgically treated adenomas. This study can serve as a benchmark for further analyses of pituitary adenoma centers in Europe.

## 1. Introduction

Pituitary adenoma surgery has evolved in recent decades because of the emergence of modern technologies such as the endoscope, neuronavigation, intraoperative magnetic resonance imaging (MRI), and intraoperative computer tomography (CT). These changes reflect a growing shift in clinical practice and bring with it new challenges to the field. There is no clear consensus regarding neurosurgical practice in Europe in the number of centers per country, caseload per center and specialized neurosurgeons, training of young neurosurgeons, etc. We created an international investigational team of neurosurgeons and performed an online survey.

## 2. Methods

The goal was to cover as many neurosurgical departments as possible in Europe that perform pituitary adenoma surgery. Each member of the study group approached their national peers with a request to participate in the survey. DN and MM were responsible for countries not covered by the other members of the research group. The study group approached 405 departments with a request to participate on the survey. The request was to complete the survey by the respective departments' chairpersons or pass it to a neurosurgeon in charge of the pituitary program. All questions used in survey are presented as supplementary data. The target was to get one completed survey per department. The survey consisted of 58 questions, which could be divided into sections:

- Demographics (countries, caseload per center, surgeons involved in pituitary surgeries, residents engaged in pituitary surgeries, and pituitary boards).
- Treatment of nonfunctioning adenomas.
- Treatment of hormone-secreting adenomas.
- Surgical techniques.

The study team was composed by neurosurgeons, and questions were answered by neurosurgeons. The study group discussed selection of questions and balance between detailed questions covering the most of pituitary surgery areas. This strategy would lead to excessively long survey with a low response rate. Therefore, the group decided to include only questions which a neurosurgeon should be ready to answer and also decided not to include case discussions. Rather, we focused on more general questions. The study period was from 1 April 2019 to 30 June 2019. This paper analyzed questions on demographics and treatment of nonfunctioning pituitary adenomas (sections A and B). The minimum time to complete the survey was 12 minutes (min) (mean time 18 min). All surveys were completed. Data from 34 European countries were obtained. We failed to obtain answers from five countries (Armenia, Kazakhstan, Kosovo, North Macedonia, and Moldova).

Altogether, 254 European Departments of Neurosurgery completed the survey. Most responses came from Germany (60), Italy (28), France (22), the UK [1], the Czech Republic [2], and Spain [3].

A univariate analysis was planned based on the following variables:

- Academic/nonacademic centers (based on participant answers).
- High-volume/low-volume centers. We defined a high-volume center as >30 pituitary adenoma surgeries per year.
- Technique applied at the center: “mainly endoscopic” (>90% of surgeries are performed endoscopically), “mainly microscopic,” and “mixed practice.”
- Regions: according to the United Nations Statistics Division [4].

The study was approved by Ethical Committee of Central Military Hospital, Prague, Czech Republic (clinical registration number 108/16–52/2021).

### 2.1. Statistics

Descriptive statistics were calculated for each survey question. Differences in responses to survey questions based on selected grouping variables were examined using Pearson's chi-square test (with a Fisher's exact test as an alternative when appropriate) with adjusted residuals. The level of statistical significance was set at *α* = 0.05. Microsoft Excel and the statistical software SPSS version 25 were used for statistical analyses and data processing.

## 3. Results

**Figure 1:**
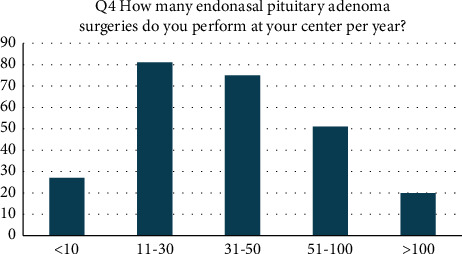
Endonasal surgeries: caseload per year.

Altogether, 254 centers participated on the survey. Academic centers represent the majority of centers in our survey (200 centers, i.e., 78.7% of the centers in the study). Total neurosurgical caseload per center (numbers pertain to general cranial and spinal neurosurgical cases): 64 centers (25.3%) performed 1500 surgeries per year and 55 (21.7%) 2000 surgeries. There were 47 centers (18.6%) that performed <1000 surgeries per year and 30 centers (11.9%) >3000 surgeries per year. Endonasal pituitary adenoma surgery volume per year: 27 centers (10.6%) treated <10 adenomas per year, 81 centers treated 11–30 adenomas per year (31.9%), 75 centers treated 31–50 adenomas per year (29.5%), 51 centers treated 51–100 adenomas per year (20%), and 20 centers (7.9%) treated >100 adenomas per year (Figure 1). All the data refer to centers involved in our survey, not to all European centers.

We defined a high-volume center if >30 adenomas are surgically treated per year, which was the case in 146 centers (57.5%). The study showed that most adenomas are treated endonasally. More than 10 transcranial surgeries for pituitary adenoma were performed in only 28 centers (11%). Typically, one or two fully trained neurosurgeons performed endonasal surgeries (one in 24.9% of the centers and two in 49.8% of the centers). More than three neurosurgeons are involved in endonasal surgery in 18 centers (7.1%).

### 3.1. Pituitary Surgery Training

**Figure 2:**
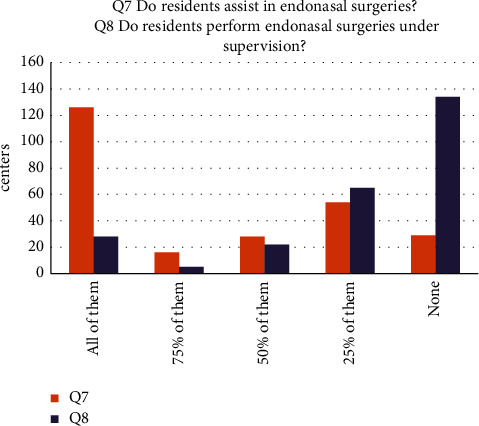
Residents' exposure to endonasal surgeries.

Concerning endonasal surgery training, the results are as follows: all residents assisted endonasal surgeries in 126 centers (49.8%); half of the residents assisted in 28 centers (11.1%) and 25% assisted in 54 centers (21.3%) during their training (Figure 2). None of the residents were exposed to endonasal surgery in 29 (11.5%) centers. In a small proportion of the centers (28, or 11%), all residents performed endonasal surgery under supervision during residency. Only one of four residents performed endonasal surgery in 65 centers (25.6%), and in 134 centers (52.8%), no resident was allowed to perform endonasal surgery.

### 3.2. Endoscopy versus Microscopy, ENT Involvement, Pituitary Board

**Figure 3:**
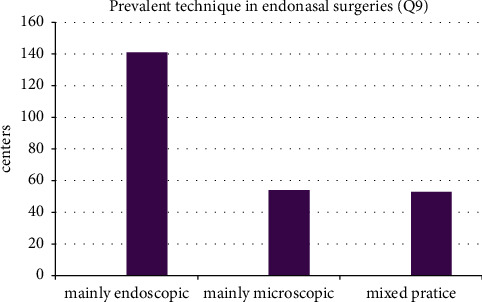
Techniques used for endonasal surgeries.

In 141 centers (56.8%), the endoscopic approach is used in >90% of the surgeries (Figure 3). The microscopic technique (>90% cases treated either microscopically or microscopically with endoscope assistance) is applied in 54 centers (21.7%). Mixed practice was noted in 53 centers (21.2%). An ENT doctor is always involved in endonasal pituitary surgery in 69 centers (27.2%), mostly in 35 centers (13.8%) and rarely or never in 127 centers (50%). A regular pituitary board (weekly or once a month) meeting was held in 147 centers (56.3%). If the board was present, every case was presented on the board in 86 centers (39.6%).

### 3.3. Clinical Scenarios in Nonfunctioning Adenomas

Nonfunctioning adenomas represent >70% of the pituitary caseload in 149 centers (58.7%), <30% of pituitary cases in 15 centers (5.9%).

**Figure 4:**
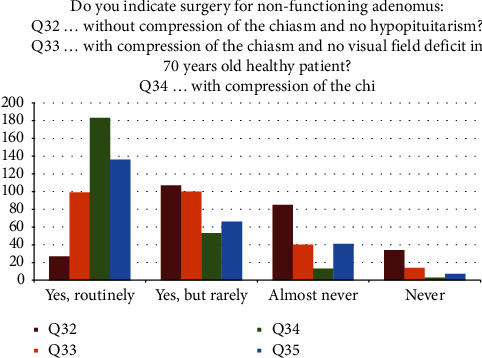
Different treatment strategies in nonfunctioning adenomas based on specific clinical.

There was a policy of no patient age limit for adenoma surgery for severe visual deficit due to adenoma in 177 centers (69.7%). No surgery was considered an option in 52 centers (20.5%) if the patient is >90 years old. An age limit of 80 years was reported in 20 centers (7.9%). The data on strategy in the treatment of nonfunctioning adenomas are summarized in Figure 4.

Surgery for nonfunctioning adenomas without compression of the chiasm and no hypopituitarism was rarely indicated in 107 centers (42.1%) and seldom or never in 119 centers (46.9%).

Surgery for nonfunctioning adenoma compressing the chiasm without a visual field deficit in 70-year-old healthy patients was routinely indicated in 99 centers (39%), rarely in 100 (39.4%) and seldom or never in 54 (21.2%).

In the same clinical scenario (but the patient is a 45-year-old healthy woman), surgery was indicated routinely in 183 centers (72%), rarely in 53 (20.9%) and seldom or never in 16 (6.3%).

In the same clinical scenario (but with a 30-year-old healthy female patient with a maternity plan), surgery was indicated routinely in 136 centers (53.5%), rarely in 66 (26%) and almost never or never in 48 (18.9%).

Radical resection of a giant adenoma was always the goal of surgery in 28 centers (11%), mostly in 133 (52.4%) and rarely or never in 30 (11.8%). The typical management strategy for a giant nonfunctioning adenoma is endonasal surgery followed by craniotomy if necessary, in 118 centers (46.5%), endonasal partial resection, then watch-and-wait for residual adenomas in 98 centers (38.6%) and craniotomy followed by endonasal surgery if necessary in 26 centers (10.2%). Combined transcranial and endonasal surgery in one session was done in four centers (1.6%).

The most typical treatments in asymptomatic residual adenoma on the first follow-up MRI were watch-and-wait in 234 centers (92.1%), upfront reoperation in 9 (3.5%), upfront radiosurgery/radiotherapy in 6 (2.4%), and dopamine agonists in 3 centers (1.2%).

### 3.4. Academic versus Nonacademic Centers

Academic centers more often performed >30 pituitary adenoma surgeries per year than nonacademic centers (66.0% versus 25.9%, *p*=0,001). More than two fully trained neurosurgeons performed endonasal surgery in 29.1% of the academic centers versus 11.1% in nonacademic centers (*p*=0.01). In 28.1% of the academic centers and 50% of nonacademic centers, <25% of the residents assisted in endonasal surgery (*p*=0.01). None of the residents performed endonasal surgery in 48% of the academic centers and 68.5% of the nonacademic centers (*p*=0.01).

No patient age limit for endonasal surgery of nonfunctioning adenoma resection causing severe visual field deficits was applied in 73.5% of the academic centers compared to 57.7% of the nonacademic (*p*=0.05). Surgery for nonfunctioning adenoma compressing the chiasm and no visual field deficit in 70-year-old healthy patients was indicated more often in academic centers (42.5% versus 26.4%, *p*=0.01). The same pattern was found in asymptomatic nonfunctioning adenoma compressing the chiasm in a 45-year-old healthy female patient (routine indication for surgery 75.5% versus 61.5%, *p*=0.05).

### 3.5. Mainly Endoscopy/Mainly Microscopy

In all, 64.8% of the centers that mainly use the endoscopic technique performed >30 pituitary adenoma surgeries per year versus 38.9% centers that primarily use microscopy (*p*=0.01). In centers using the endoscopic approach, transcranial pituitary adenoma surgery was performed less often (0–2 transcranial surgeries, 59.2% versus 44.4%, *p*=0.05). In centers using endoscopy, an ENT surgeon is more often included in the surgical team (always or mostly, 56.3% versus 11.1%, *p*=0.001). Pituitary board is more often held in centers using endoscopy (66.2% versus 35.2%, *p*=0.001).

### 3.6. High-Versus Low-Volume Centers

In 38.9% of low-volume centers, only one fully trained neurosurgeon was involved in pituitary surgery. In contrast, in 14.5% of high-volume centers, only one fully trained neurosurgeons took part in pituitary surgery (*p*=0.001). Totally, 69.4% of the residents in low-volume centers never performed adenoma surgery; in high-volume centers, 40.4% of the residents never performed adenoma surgery (*p*=0,001). In 38.9% of the low-volume centers, an ENT doctor is never on the surgical team. An ENT doctor is on the surgical team in 25.3% of the cases in high-volume centers (*p*=0.001). Pituitary board meetings were held in 71.2% of the high-volume centers versus 36.1% of the low-volume centers (*p*=0.001).

### 3.7. Regions

None of the residents performs endonasal surgery under supervision in 72.1% of eastern centers and 38.6% of northern centers (*p*=0.01). An ENT doctor was never a member of the surgical team in 45.2% of the western centers; an ENT doctor was on the surgical team in 15.7% in northern centers (*p*=0.001). Routine pituitary board meetings were held in 67.1% of the northern countries and 23.3% of the centers in eastern countries (*p*=0.001). There was no age limit for nonfunctioning adenoma resection causing severe visual field deficits in 81.4% of the Western centers and 51.4% of the northern centers (*p*=0.001). Surgery for nonfunctioning adenoma asymptomatically compressing the chiasm in a 30-year-old healthy woman with maternity plans was almost never indicated in 35.1% of the southern centers and 9.8% of the Western centers (*p*=0.01).

## 4. Discussion

To our knowledge, this survey on pituitary practice is the largest neurosurgical survey of its kind. Solari et al. presented an excellent survey on pituitary surgery in Italy [5]. They received data from 37 centers out of 41 where pituitary surgery is performed. Endoscopy was dominant in transsphenoidal approach (1204 cases performed endoscopically, 53 microscopically, and 53 endoscope-assisted microscopic). A multidisciplinary tumor board was convened regularly in 32 of 37 centers. Solari et al. achieved a very high response rate in Italian pituitary survey. Our group analyzed data from 254 departments around Europe, and the absolute number is much higher but relative coverage is obviously lower than in a single country study. Thus, data from our study should be analyzed cautiously. All members of our study team were responsible for their regions, and DN and MM were responsible to the countries not covered by study members. Nachtigall et al. conducted a survey study on timing of MRI scanning and awareness of gadolinium retention after repeated MRI scanning of pituitary tumors [6]. DeDivitis et al. conducted a survey focusing on the role of endoscopy in transsphenoidal surgery [7]. The authors distributed a web-based multi-item questionnaire to 393 neurosurgical centers employing an invitation e-mail. Complete questionnaires were available for analysis from 87 centers. The findings revealed that the endoscope was used in 85.2% of the transsphenoidal procedures and the microscope in 14.8%. There is an ongoing debate on the centralization of care and centers of pituitary excellence [8, 9]. One discussed topic was the volume of pituitary adenomas treated per year per unit and per neurosurgeon. Some papers advocate 50 surgeries per neurosurgeon per year [8, 10]. Barker et al. examined the volume-outcome relationship for transsphenoidal pituitary tumor surgery using the US Nationwide Inpatient Sample in 1996–2000 [11]. The authors define a high-volume center as performing a minimum of 25 cases per year. Already in 1997, Ciric et al. reported a correlation between the experience of the pituitary surgeon and complications of transsphenoidal surgery based on three sources: a national survey, review of the literature, and personal experience [12]. Casanueva et al. discussed the scenario of a single dedicated pituitary neurosurgeon per center and addressed the drawbacks of such an approach [8]. The benchmark of 50 cases per year and neurosurgeon and a minimum of two pituitary neurosurgeons per department were discussed. This scenario would result in a minimum of 100 cases per year and department. In our survey, only 7.9% of the centers performed >100 adenoma surgeries per year. In our analysis, we used a very low threshold for a high caseload per center, i.e., 30 cases/year. Still, 42.5% of the centers in our survey did not fulfill this weak criterion. DeDivitis et al. documented in their survey that the transcranial approach is used predominantly for suprasellar tumors that lack significant intrasellar portions [7]. This finding corresponds to our results, where we found that 0–2 transcranial surgeries for pituitary adenoma per year were performed in 52.8% of the centers.

In most centers, the residency program provides limited experience in transsphenoidal pituitary surgery. Our study confirms these findings: only in half of the centers, all residents assisted in pituitary surgery during their training. In more than half of the centers, not a single resident performs a pituitary surgery under supervision. This situation does not allow the graduate to gather enough experience to practice independently immediately after completing training [9]. McLaughlin et al. proposed strict criteria that define a pituitary center of excellence with particular emphasis on 3 key areas: experienced, multidisciplinary patient care; postgraduate medical education; and focused research endeavors [9]. Casanueva et al. proposed three recommendations after completing residence: [4] completion of a formal postgraduate fellowship in pituitary surgery, [5] completion of a postgraduate fellowship in skull base or neuro-oncologic surgery at a high-volume pituitary center, or [6] completion of postgraduate subspecialty training at a high-volume pituitary center [8].

According to DeDivitis et al., endoscopy is a preferred surgical mode for pituitary adenomas [7]. The endoscope was used in 85.2% of the transsphenoidal procedures, while the microscope was used in 14.8%. In this study, a combination of techniques was not analyzed. In our survey, endoscopy was the primary technique in 56.8% of the centers, but mixed practice was also frequent (21.2%). The involvement of an ENT doctor in surgery for pituitary adenoma has often been discussed. Many studies documented that teamwork is recommended for skull base pathologies [3]. Based on our results, an ENT doctor is rarely or never involved in pituitary surgery in 50% of the study centers. Obviously, the role of an ENT specialist is more relevant in centers using the endoscopic technique.

Wheless et al. documented the clinical impact of a multidisciplinary head and neck tumor board [13]. A pituitary board should be held in every Pituitary Tumor Center of Excellence [8]. Still, the regular pituitary board was held in only 56.3% of the centers. On the other hand, there may be informal pituitary boards in high-volume centers where complex cases are discussed. These complex cases may also involve closer neurosurgical and ENT cooperation.

Surgery for nonfunctioning macroadenoma without compression of the chiasm and no hypopituitarism was rarely or never indicated in 90% of the centers. This observation is in line with general pituitary adenoma recommendations [2, 14]. Already in 1990, Reincke et al. reported a study on incidentalomas [15]. They described 18 patients with an intrasellar mass incidentally discovered by CT or MRI. The average size of the mass was 13 mm (range 5–25 mm). They concluded that the “incidentaloma” of the pituitary gland is a benign condition that does not necessarily require neurosurgical intervention.

Dekkers et al. analyzed evidence for treatment and follow-up for nonfunctioning adenomas [16]. They found only observational studies and concluded that incidentalomas, although benign in nature, need individualized treatment and lifelong radiological and endocrinological follow-up. According to a review by Murad et al., the surgical risks for nonfunctioning pituitary adenomas include 1) surgical death in 1%, 2) cerebrospinal fluid leakage/fistula in 3%, meningitis in 1%, 3) transient diabetes insipidus in 11%, 4) persistent diabetes insipidus in 5%, 5) new anterior pituitary deficits in 9%, and 6) new visual field defects in 3% [1]. The risk of surgical complications, including postoperative hypopituitarism, should guide surgical treatment decisions in an asymptomatic patient with pituitary adenoma. Our study found that 39% of the centers routinely indicate surgery in 70-year-olds and healthy patients with adenoma compressing the chiasm without visual field deficit, and 72% would recommend surgery if the patient is a 45-year-old healthy woman. Fewer centers (53.5%) would recommend surgery in 30-year-old healthy women with a maternity plan. According to Fatemi et al., the risk of postoperative hypopituitarism is approximately 5% [17]. This risk should be balanced with the risk of apoplexy during pregnancy [18]. As always in preventive surgery, an opinion of a well-informed patient is of utmost importance. Surprisingly, 63.4% of the centers in our review set radical resection for giant adenoma as the primary goal of surgery. According to Iglesias et al., radical resection is achieved in 14.7–41.2% of giant adenomas [19]. Chabot et al. analyzed results in 39 consecutive large or giant adenomas [20]. Gross total resection of the pituitary macroadenoma was achieved in 56.4% of the cases based on postoperative MRI.

Combined simultaneous endonasal and transcranial approaches for giant adenomas were reported [21–23]. In our survey, only 1.6% of the centers are currently using this technique. Even-Zohar et al. suggest that dopamine agonist treatment should be routinely considered for managing incompletely resected nonfunctioning adenomas [24]. They assume that dopamine agonist treatment may prevent residual tumor enlargement. According to our survey, this strategy is applied in 1.2% of centers. On the other hand, this approach is not generally accepted [25]. Sheehan et al., on behalf of the Congress of Neurological Surgeons, published a systematic review and evidence-based guidelines for managing patients with residual or recurrent nonfunctioning pituitary adenomas [25]. They recommend serial neuroimaging in patients with small residual tumors. European centers mostly apply a watch-and-wait strategy (92.1% of the centers) and seldomly (2.4%) implement upfront radiosurgery/radiotherapy.

### 4.1. Academic versus Nonacademic Centers

Academic centers are more active in indicating pituitary surgery. For instance, they often have no age limit for endonasal surgery of nonfunctioning adenoma resection, causing severe visual field deficit (73.5% versus 57.7%). In addition, they more often indicate pituitary surgery of non-functioning adenoma compressing the chiasm without visual field deficit (42.5% versus 26.4%).

### 4.2. Mainly Endoscopy/Mainly Microscopy

In all, 64.8% of the centers mostly used the endoscopic technique. Endoscopy is more often used in centers performing >30 pituitary adenoma surgeries per year than in low-volume centers (64.8% versus 38.9%). Younus et al. showed a continuous learning curve of endoscopic skull base surgery even after 1000 cases [26]. The learning curve may explain why endoscopy is used less often in low-volume centers. Endoscopy is more often performed in the presence of ENT surgeons [3]. Our survey found that an ENT surgeon is more often included in the surgical team (always or mostly: 56.3% versus 11.1%).

### 4.3. High-Versus Low-Volume Centers in Relation to Adenoma Surgery

In high-volume centers, residents are more often included in pituitary surgery (40.4% versus 69.4%). The pituitary board was mostly held in high-volume centers (71.2% versus 36.1% in low-volume centers).

### 4.4. Regions

In eastern countries, residents less often participate in pituitary surgery compared to northern countries. In northern countries, ENT doctors are more often a surgical team member than in western countries (45.2% versus 15.7%). The routine pituitary board is more frequent in northern than eastern countries (67.1% versus 23.3%).

### 4.5. Strength and Limitations

The main limitations of our study are related to study design. Every survey suffers from sampling bias that largely depends on the response rate. The strength of our survey is the large sample of data with a high response rate and 100% completion rate. It should be emphasized that each respondent in our survey represents one neurosurgical center.

## 5. Conclusion

The most centers included in our survey cannot be considered as high-volume centers. In most centers, one or two neurosurgeons perform all the pituitary adenoma surgeries. We also found that residents gain limited exposure to this type of surgery. Surprisingly, the formal pituitary board is not a standard. The involvement of an ENT doctor in pituitary surgery is limited. Nonfunctioning adenomas represent most of treated adenomas in most centers. Differences in pituitary adenoma practice were observed between low-volume centers, centers using endoscopy or microscopy, academic and nonacademic centers and regions in Europe. This study may serve as a benchmark for further analyses of pituitary adenoma centers in Europe and as a recommendation to centralize pituitary adenoma care.

## Data Availability

The data used to support the findings of this study are available from the corresponding author upon request.

## Supplementary Materials

Supplementary Materials Supplementary data: all 58 questions used in the European Pituitary Adenoma Surgery Survey (EU-PASS).
