# Supplementary material for: Pituitary Adenoma Surgery Survey: Neurosurgical Centers and Pituitary Adenomas
Source: Int J Endocrinol. 2022 Apr 11;2022:7206713. doi: 10.1155/2022/7206713 (PMC9017568; doi:10.1155/2022/7206713)
Supplement: Supplementary Materials — Supplementary data: all 58 questions used in the European Pituitary Adenoma Surgery Survey (EU-PASS). [file 7206713.f1.docx]

EUropean Pituitary Adenoma Surgery Survey: EU-PASS

Questions:

1. Which country are you currently working in?
2. Are you at an academic center?

Yes

No

1. What is the total number of operations performed yearly in your center?

1000

1500

2000

3000

>3000

1. How many endonasal pituitary adenoma surgeries do you perform at your center per year?

≤10

11-30

31-50

51-100

>101

1. How many transcranial pituitary adenoma surgeries do you perform at your center per year?

0-2

3-5

6-9

10-15

≥15

1. How many fully trained neurosurgeons perform endonasal surgeries?

1

2

3

4

5

>6

1. Do residents assist in endonasal surgeries?

All of them

75% of them

50% of them

25% of them

None

1. Are residents doing endonasal surgeries under supervision during their residency?

All of them

75% of them

50% of them

25% of them

None

1. What percent of your endonasal cases that has undergone the following types of surgery?

Microscopic %

Microscopic + endoscope-assisted %

Endoscopic %

1. Do you perform the surgeries with an ENT specialist?

Always

Mostly

Sometimes

Rarely

Never

1. For which type of cases are you working together with an ENT doctor?

Pituitary adenomas

Craniopharyngiomas

Meningiomas

Chordomas/chondrosarcomas

Transcribriform for malignancies

1. Do you have regular pituitary board meetings with an endocrinologist, radiologist, radiosurgeon, etc?

Every week

Every month

Sporadically

Never

1. If you have these meetings, do you discuss

Every case

Selected (challenging) cases only

**Prolactinoma**

1. How many prolactinomas do you treat surgically per year?

1-5

6-10

11-20

21-30

>31

1. Do you ever consider surgery as the primary treatment modality for prolactinomas?

No

Yes (please specify the indication…)

1. What is the time between consultation and surgery for apoplexy on medication, severe headaches and moderate visual field deficit?

The same day

Within 48 hours

Within 7 days

After 7 days

First watch and wait and medical therapy, then a decision is made

**Acromegaly**

1. How many cases of acromegaly do you treat per year?

1-5

6-10

11-20

21-30

>31

1. What is the percent of GH secreting microadenomas which are first treated pharmacologically and surgery is indicated as a second line treatment among all GH secreting microadenomas that you surgically treat?

Pretreated cases %

1. What is the percent of pharmacologically pretreated macroadenomas?

Pretreated cases %

1. Which medications do you use for pretreatment?

Dopamine agonists

First-generation somatostatin analogues (lanreotide and octreotide, etc.)

Second-generation somatostatin analogues (pasireotide, etc.)

Growth hormone receptor antagonist (pegvisomant)

1. Do you inform your team and your patient whether a hormonal remission is a realistic goal of the surgery beforehand?

Always

Mostly

Sometimes

Rarely

Never

1. Do you consider debulking surgery for an invasive growth of the tumor, where hormonal remission is not a realistic goal of the surgery and the patient has no visual deficit?

Always

Mostly

Sometimes

Rarely

Never

**Cushing‘s disease**

1. How many cases of Cushing’s disease do you treat per year?

1-5

6-10

11-20

21-30

>31

1. What is the percent of pharmacologically pretreated cases (ketoconazole, metyrapone, etc.)

Pretreated cases %

1. What is the goal of pretreatment?

Safer anaesthesia

Better postoperative healing

Lower risks of postoperative medical complications

To overcome the waiting period for surgery

Other (please specify)

NA

1. What is the percent of patients with a preoperative dynamic MRI?

%

1. What is the percent of patients with a preoperative 7 Tesla MRI?

%

1. What is the percent of cases with catheterization and petrous sinus blood sampling?

%

**Non-functioning adenomas**

1. What is the percent of non-functioning versus hormone-secreting adenomas in your service?

Non-functioning adenomas %

Hormone-secreting adenomas %

1. Do you have any age limit for non-functioning adenoma resection for severe visual field deficit due to adenoma?

None

>90

>80

>70

1. Do you indicate surgery for non-functioning adenomas without compression of the chiasm and no hypopituitarism?

Yes, routinely

Yes, but rarely

Almost never

Never

1. Do you indicate surgery for non-functioning adenomas with compression of the chiasm and no visual field deficit in 70 years old healthy patient?

Yes, routinely

Yes, but rarely

Almost never

Never

1. Do you indicate surgery for non-functioning adenomas with compression of the chiasm and no visual field deficit in 45 years old healthy woman?

Yes, routinely

Yes, but rarely

Almost never

Never

1. Do you indicate surgery for non-functioning adenomas with compression of the chiasm and no visual field deficit in 30 years old healthy woman with maternity plans?

Yes, routinely

Yes, but rarely

Almost never

Never

1. For giant pituitary adenoma (>40 mm), is complete resection your main goal?

Always

Mostly

Sometimes

Rarely

Never

1. For giant non-functioning adenomas, what is your typical management strategy?

Endonasal followed by craniotomy if necessary

Craniotomy followed by endonasal if necessary

Combining craniotomy with the endonasal approach in one session

Endonasal partial resection, then watch and wait for residual adenomas (whenever possible due to the clinical condition)

Endonasal partial resection + upfront radiosurgery or iMRT

1. What is the median length of hospital stays for giant adenomas?

<5 days

5-10 days

>10 days

1. What is the typical treatment strategy in asymptomatic residual adenoma on the first follow-up MRI?

Upfront radiosurgery/radiotherapy

Upfront reoperation

Watch and wait

Dopamine agonists

**Postoperative medication**

1. Do you routinely prescribe hydrocortisone after surgery or do you follow cortisol levels in the early postoperative period and offer a substitution for new hypocorticalism only?

Routine dosage of hydrocortisone substitution

According to postoperative cortisol levels (done by a neurosurgeon)

According to postoperative cortisol levels (done by endocrinologist)

1. Do you consider reoperation in Cushing`s disease if the early postoperative cortisol levels are above the normal range?

Yes

No

**Technique in pituitary adenoma surgery**

1. How do you close the sella in cases without intraoperative CSF leakage (multiple answers permitted)?

We don’t close the sella

Fat

Fascia

Tissue glue

Bone

Dural substitutes

Collagen sponge coated with coagulation factors

Nasoseptal flap

Others (Medpore, middle turbinate free flap, etc.)

1. How do you close the sella in cases with intraoperative CSF leakage (multiple answers permitted)?

Fat

Fascia

Tissue glue

Bone

Dural substitutes

Collagen sponge coated with coagulation factors

Nasoseptal flap

Others (Medpore, middle turbinate free flap, etc.)

1. Do you use fluorescein in standard pituitary surgery to detect intraoperative leaks?

Yes

No

1. How often do you insert a lumbar drain immediately after pituitary surgery in case of intraoperative CSF leak?

Always

Mostly

Sometimes

Rarely

Never

1. If yes, how many days do you leave the lumbar drain open?

1-3

4-5

≥6

1. Do you administer antibiotics in inserted lumbar drains?

Never

Case by case

Always

1. For suspected postoperative CSF leaks, do you base your decision on?

clinical examination

Beta trace protein

Beta transferrin

CT cisternography

1. For postoperative low flow CSF leaks do you

Indicate reoperation always on the day of diagnosis

Indicate reoperation always, but surgery may be postponed for 1-2 days

Insert a lumbar drain + bed rest

Insert a lumbar drain + no bed rest

Bed rest only

1. How often do you perform expanded approaches for pituitary adenomas?

% of your pituitary cases

1. How often do you use nasoseptal or other pedicled flaps in these cases?

Mostly

Sometimes

Rarely

Never

1. What is the percent of cases with early MRI (1-3 postoperative days)?

Routinely

For selected cases

Never

1. How often do you use intraoperative MRI (iMRI) in pituitary adenoma surgery?

iMRI is not available

iMRI is available and used almost always

iMRI is available and used for selected cases

iMRI is available, but not used for pituitary adenomas

1. How often do you use intraoperative CT (iCT) in pituitary adenoma surgery?

iCT is not available

iCT is available and used almost always

iCT is available and used on selected cases

iCT is available, but not used for pituitary adenomas

1. Do you systematically assess anosmia?

Routinely

For selected cases only

No

1. Do you routinely assess nasal morbidity?

Yes, clinical examination by a neurosurgeon

Yes, clinical examination by an ENT doctor

Yes, clinical examination and by quality of life questionnaires

No

1. Do you personally follow-up the patient?

Yes

No

1. For how long?

For ever

First 5 years

First year

First follow-up control

No

1. When do you perform the first follow-up scan?

1 month

2 months

3-4 months

5 months

>5 months
